# Uncovering the involvement of DoDELLA1-interacting proteins in development by characterizing the DoDELLA gene family in *Dendrobium officinale*

**DOI:** 10.1186/s12870-023-04099-w

**Published:** 2023-02-13

**Authors:** Danqi Zeng, Can Si, Jaime A. Teixeira da Silva, Hongyu Shi, Jing Chen, Lei Huang, Juan Duan, Chunmei He

**Affiliations:** 1 Key Laboratory of South China Agricultural Plant Molecular Analysis and Genetic Improvement, Provincial Key Laboratory of Applied Botany, South China Botanical Garden, Chinese Academy of Sciences, Guangzhou, 510650 China; 2 South China National Botanical Garden, Guangzhou, 510650 China; 3 University of the Chinese Academy of Sciences, Beijing, 100049 China; 4 Independent Researcher, Ikenobe 3011-2, Miki-cho, Kagawa-Ken 761-0799 Japan

**Keywords:** *Dendrobium officinale*, *DoDELLA* genes, Gene expression, Yeast two-hybrid library screening assay

## Abstract

**Background:**

Gibberellins (GAs) are widely involved in plant growth and development. DELLA proteins are key regulators of plant development and a negative regulatory factor of GA. *Dendrobium officinale* is a valuable traditional Chinese medicine, but little is known about *D. officinale* DELLA proteins. Assessing the function of *D. officinale* DELLA proteins would provide an understanding of their roles in this orchid’s development.

**Results:**

In this study, the *D. officinale* DELLA gene family was identified. The function of DoDELLA1 was analyzed in detail. qRT-PCR analysis showed that the expression levels of all *DoDELLA* genes were significantly up-regulated in multiple shoots and GA_3_-treated leaves. *DoDELLA1* and *DoDELLA3* were significantly up-regulated in response to salt stress but were significantly down-regulated under drought stress. DoDELLA1 was localized in the nucleus. A strong interaction was observed between DoDELLA1 and DoMYB39 or DoMYB308, but a weak interaction with DoWAT1.

**Conclusions:**

In *D. officinale*, a developmental regulatory network involves a close link between DELLA and other key proteins in this orchid’s life cycle. DELLA plays a crucial role in *D. officinale* development.

**Supplementary Information:**

The online version contains supplementary material available at 10.1186/s12870-023-04099-w.

## Background

Gibberellins (GAs) are a class of vital plant phytohormones that play pivotal roles in various processes associated with plant growth and development, ranging from cellular functions to organ development, and encompassing wide processes such as cell elongation, seed germination, leaf expansion, flowering and pollen maturation [1–4]. GAs are also involved in plants’ transformation from vegetative to reproductive growth [5]. Already 40 years ago, GA derived from the pathogenic fungus *Gibberella fujikuroi* caused the over-elongation of rice (*Oryza sativa* L.) [6]. There are two classes of GA, those with biological activity and non-bioactive forms. The major bioactive GAs include GA_1_, gibberellic acid (GA_3_), GA_4_ and GA_7_ [1], which have been intensively studied. In the model plants *Arabidopsis thaliana* and rice, most of the components of the GA signaling pathway have been identified over the past decade, with key components including the GA receptor, DELLA growth inhibitors, and F-box proteins [2]. *GA-INSENSITIVE DWARF1* (*GID1*) was first identified as a GA receptor in rice [7]. In *A. thaliana*, the major GA receptors were recognized as *GID1a*, *GID1b*, and *GID1c*, *GID1* homologs with redundant or overlapping functions and different responses to GA [8–10]. Strong evidence supported the notion that the endpoint of GA signaling is the regulation of transcriptional activity [11, 12]. Interestingly, GA is closely related to DELLAs, a small group of nuclear proteins, and the GA receptor mediates GA-induced DELLA proteolysis [13]. The current model to explain the action of GA proposes that DELLA protein inhibits plant growth whereas GA signals promote plant growth by overcoming DELLA-mediated growth inhibition [2, 14]. In other words, and simplistically speaking, DELLA is considered to be a negative regulator of GA [15–17]. *GID1* receptors communicate with DELLA proteins in a DELLA-dependent manner, leading to their degradation via a ubiquitin–proteasome pathway [8, 9, 18]. Furthermore, the GA-GID1-DELLA complex is targeted by F-box proteins, typically GID2 in rice and SLEEPY1 in *A. thaliana*, causing proteasome-mediated degradation [8, 19].

DELLA proteins are a subset of putative transcription regulators of the plant-specific GAI-RGA-and-SCR (GRAS) family [20]. DELLA proteins have a short stretch of five amino acids (D-E-L-L-A) that is strictly conserved in all plant species, hence the name of this transcription factor (TF). In addition, DELLA proteins also have conservative motifs such as the Asp-Glu-Leu-Leu-Ala motif, the VHYPN domain, a poly (serine/threonine) stretch and two leucine heptads repeats, the latter mediating protein–protein interactions [3]. DELLA proteins, as TFs, regulate plant growth and development via a complex mechanism. For example, the *A. thaliana* DELLA protein RGL2 can act as a major repressor in the control of seed germination [21–23]. In addition to RGL2, *A. thaliana* also has four other DELLA members, GAI, RGA, RGL1 and RGL3, which participate in various processes associated with growth and development, such as flowering, as well as stem and root development [16, 22, 24, 25]. However, there is only a single DELLA protein in rice, namely SLENDER RICE1 (SLR1) [17, 26]. SLR1, which was shown to control seedling growth and stem elongation, was used in hybrid rice breeding [27–29]. Similarly, tomato (*Lycopersicon esculentum* L.) also contains only one DELLA protein PROCERA, which affects many aspects of tomato growth, such as fruit development, seed and leaf development, stomatal closure of guard cells, and other processes [30–33]. DELLA proteins can integrate the responses of different plant hormones in response to signals emitted during adverse environmental conditions, allowing plants to adapt and cope with associated stresses [34].

As was briefly mentioned above, DELLA proteins are plant nuclear factors that regulate gene expression, exerting their inhibitory effect on GA signal transduction. DELLA proteins have dual functions, by adjusting downstream genes to modulate plant development, and also allowing GA to regulate a variety of developmental processes [4]. DELLA proteins are key integrators of hormone signals by directing or binding other transcriptional regulators to inhibit cell expansion and proliferation [30]. DELLA proteins might also interact with other TFs, thereby suppressing their DNA binding and transcriptional activity [20]. In *A. thaliana*, the DELLA protein RGA interacts with PHYTOCHROME INTERACTING FACTOR 3 and 4 (PIF3 and PIF4) and blocks their transcription activity, leading to shortening of the hypocotyl [35, 36]. PIF3 and PIF4, which belong to the bHLH (basic-helix–loop–helix) TF family, harbor a conserved bHLH domain [37]. PIF3 and PIF4 are also negatively regulated by DELLA proteins. In rice, the DELLA protein SLR1 and GA signaling are genetically related to cellulose synthesis [38]. Initially, SLR1 directly interacts with NACs (NAC29 and NAC31, which activate the expression of MYB61 and CELLULOSE SYNTHASE genes), following which GAs initiate the repression of NACs by triggering the proteasomal degradation of SLR1, and finally promoting cellulose biosynthesis [38]. DELLA proteins’ functions are influential and widespread, explaining why they may interact with many TFs [3, 39]. As a result of their interaction with different families of TFs, DELLA proteins are able to transmit information to various transcriptional circuits that control plant growth and development [40], suggesting that they are a central hub in plant development. DELLA proteins, which interact with TFs that are bound to their target genes, can be detected by chromatin immunoprecipitation (ChIP) using tagged DELLA [40].

Over the past two to three decades, molecular and genetic studies have helped to decipher the cellular events responsible for GA activities in some model plants such as *A. thaliana* and rice, and this has opened the door to GA research in other plant species. In *A. thaliana*, DELLA regulates the development of floral organs [25], and this process is regulated by a variety of plant hormones and stress signaling pathways [13]. In rice, the DELLA protein SLR1 regulates miR396 to control cell proliferation [28] whereas GAs promote plant elongation, mainly by affecting cell proliferation and cell elongation [41], further implying a close association between DELLA and GAs. In barley (*Hordeum vulgare* L.), the role of the conservative *DELLA* gene is to control stem meristems and the inflorescence meristem [42], which corresponds to the roles of *A. thaliana DELLA* genes *GAI* and *RGA* in regulating the growth of stem and inflorescence meristems [43]. In tomato (*Solanum lycopersicum* L.), *SlDELLA* plays a role in the transformation between ovary and fruit as well as in the growth of other reproductive structures such as style elongation and stigma development [30]. Additionally, as part of GA signaling, a DELLA protein regulated leaf bud dormancy in peach (*Prunus persica* (L.) Batsch) via a GA-GID1-DELLA module.

Even though there has been considerable progress in understanding the function of DELLA proteins in model plants, particularly *A. thaliana*, their biological functions and regulatory mechanisms in non-model plants are largely unknown. *Dendrobium officinale* is a traditional Chinese medicinal plant with considerable medicinal and market value, but its production relies on breakthrough research related to growth and development. The mechanism by which DELLA proteins regulate growth and development in *D. officinale* are unknown and unclear. In this study, we identified the *D. officinale* DELLA gene family, which consists of four DELLA proteins (DoDELLA1, 2, 3 and 4). DoDELLA1 was selected for functional analysis. This study provides vital information regarding the involvement of DELLA proteins in the development of this orchid, which may allow for targeted regulation of plant production and management.

## Results

### Identification and analysis of *DoDELLA* gene family members in *D. officinale*

**Fig. 1:**
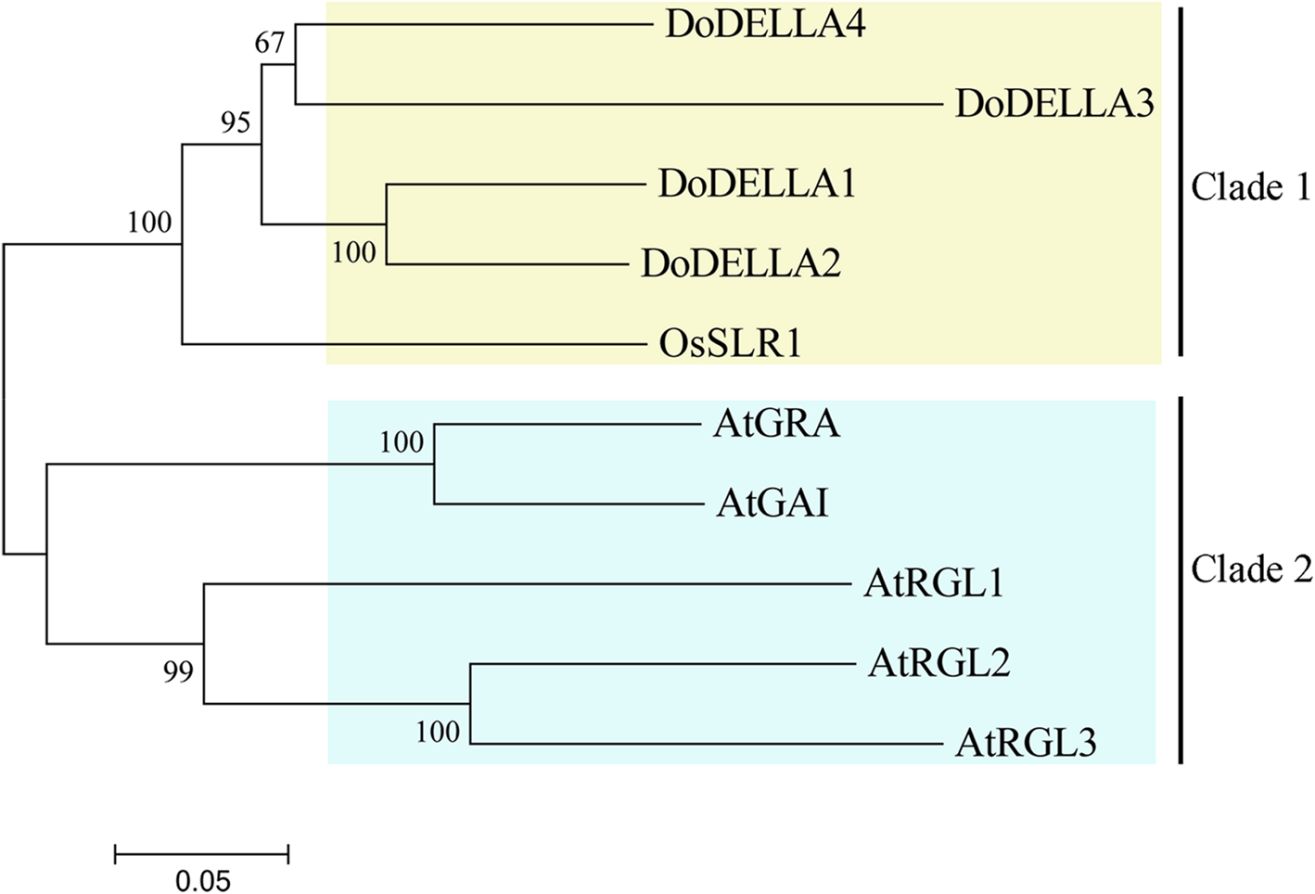
Phylogenetic analysis of DELLA proteins from *Dendrobium officinale* (Do) , *Oryza sativa* (Os) and *Arabidopsis thaliana* (At). The neighbor-joining (NJ) phylogenetic tree was constructed by MEGA 7 software with 1000 bootstrap replications. Only bootstrap values higher than 50% are shown

Using bioinformatic methods, a total of four *DoDELLA* genes were identified in the *D. officinale* genome database. To study the evolutionary patterns of these four DELLA proteins, an unrooted phylogenetic tree was constructed by aligning the full-length DELLA protein sequences of four candidate DoDELLA proteins (DoDELLA1, 2, 3 and 4), one OsDELLA protein from *O. sativa* (OsSLR1), and five AtDELLA proteins from *A. thaliana* (AtGRA, AtGAI, AtRGL1, 2 and 3). In our phylogenetic tree, clade 1 contained *D. officinale* and the *O. sativa* (monocotyledonous plant) DELLA protein while clade 2 contained the *A. thaliana* (dicotyledonous plant) DELLA proteins (Fig. 1). DoDELLA1 and 2 had a strong bootstrap value (100%) (Fig. 1), potentially indicating their similar or overlapping functions.

### Multiple sequence alignment analysis of DoDELLA proteins

**Fig. 2:**
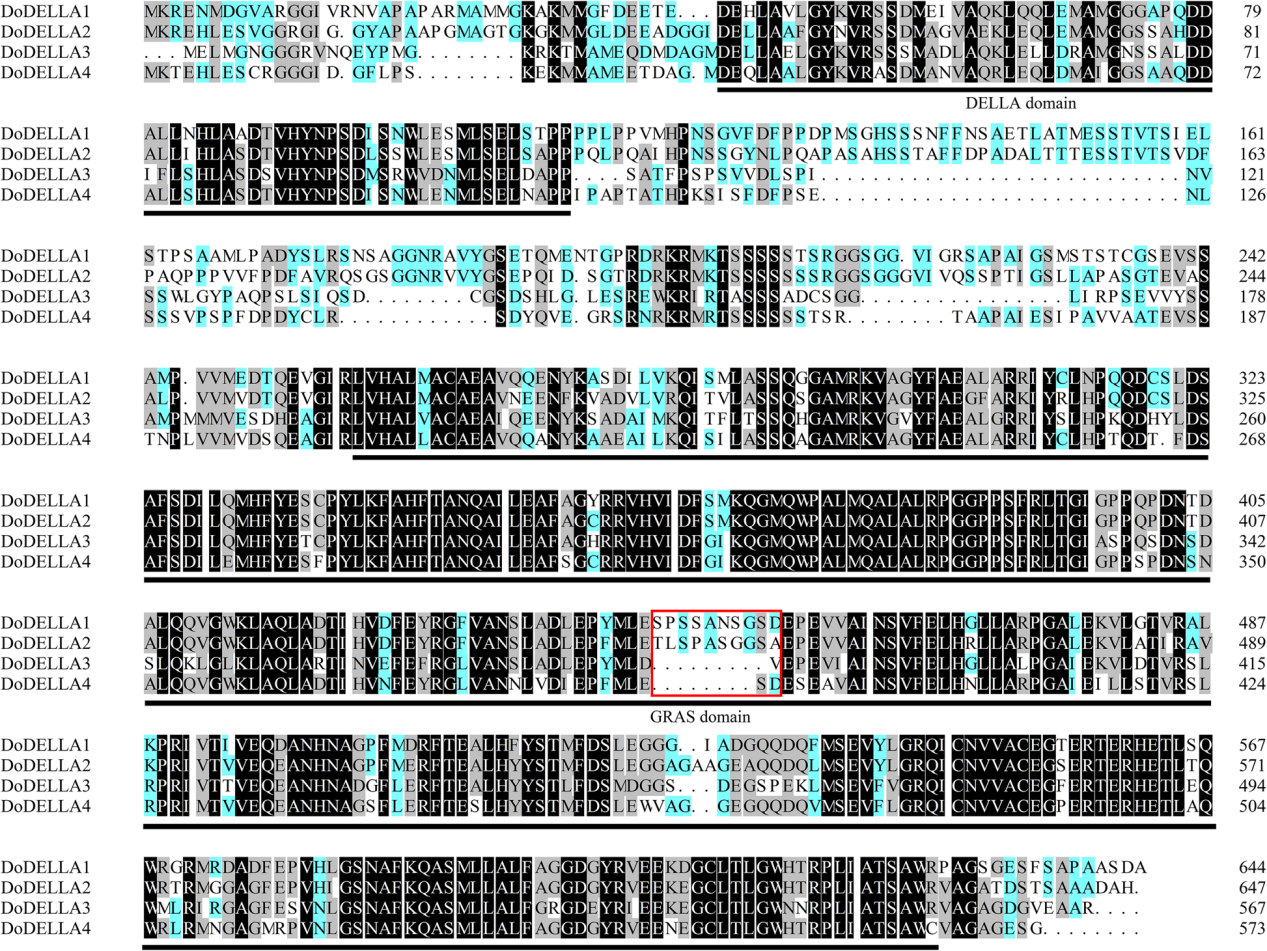
Sequence alignment of four DoDELLA proteins displaying ≥ 50% homology. DNAMAN 8.0 software was used. Different colors indicate different levels of conservation in residues. Black, gray and green indicate 100%, 75%, and 50% identical residues, respectively. The DELLA and GRAS domains are underlined

The protein structural domains of the *D. officinale* DELLA family members were identified by subjecting them to multiple sequence alignment analysis. A previous study showed that DELLA proteins usually contain two highly conserved domains, the DELLA domain and the GRAS domain [24]. As expected, all four DoDELLA proteins have highly conserved domains, i.e., the N-terminal DELLA domain (including the TVHYNP/VHYNP domain) and the C-terminal GRAS domain (Fig. 2). Compared to DoDELLA3 and 4, the DoDELLA1 and 2 protein sequences showed a higher percentage of amino acid similarity (Fig. 2, indicated by a red box), which was consistent with the results of the phylogenetic tree (Fig. 1). The DELLA and GRAS domains were conserved in all four DoDELLA proteins but their amino acid sequences were relatively different.

### Expression analysis of *DoDELLA* genes at different developmental stages

**Fig. 3:**
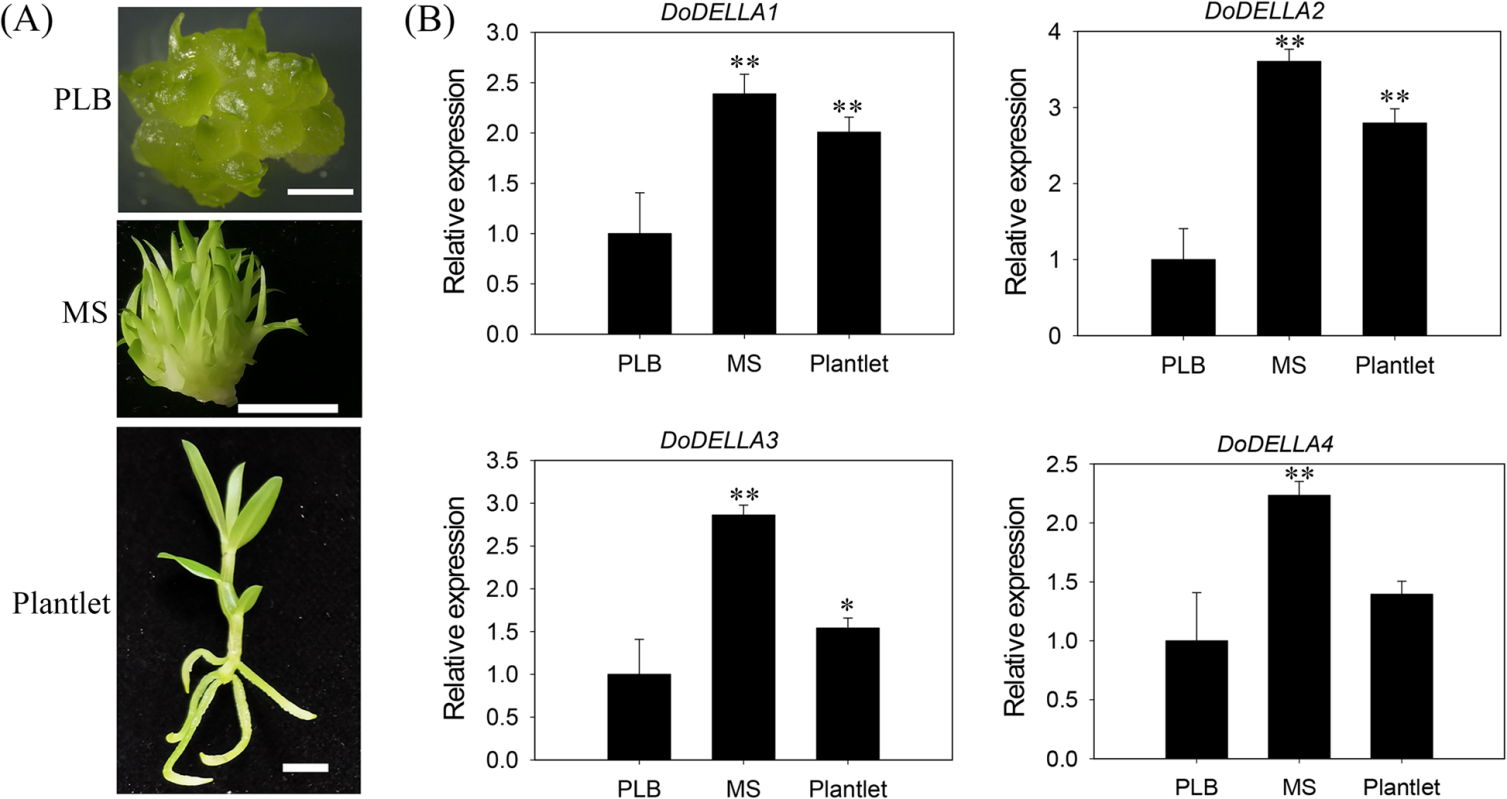
Expression analysis, using qRT-PCR, of *Dendrobium officinale DoDELLA* genes in different organs (PLB: protocorm-like body; MS: multiple shoot; plantlet), corresponding to different stages of development. **A** Three organs analyzed. Bars: 2 mm for PLB; 1 cm for MS and plantlet. **B** Expression pattern of four *DoDELLA* genes in the three organs. Data indicates the mean ± SD of three biological replicates (*n* = 3). Asterisks denote statistically significant differences: * *p* < 0.05; ** *p* < 0.01

The expression pattern rather than molecular activity endows *DELLA* genes with their functional specificity [44]. Since *DELLA* genes tend to be associated with plant development, we analyzed the changes in expression of the four *DoDELLA* genes in protocorm-like bodies (PLBs), multiple shoots (MSs, i.e., without roots) and plantlets (about 5 cm tall). Highest expression was observed for all *DoDELLA* genes in MSs, significantly more than in PLBs, and significantly more than in plantlets for *DoDELLA1* and* 2* (Fig. 3). Thus, *DoDELLA1*-*4* likely play a role in the development of one or more of these organs.

### Expression analysis of *DoDELLA* genes in response to GA_3_ and two abiotic stresses

**Fig. 4:**
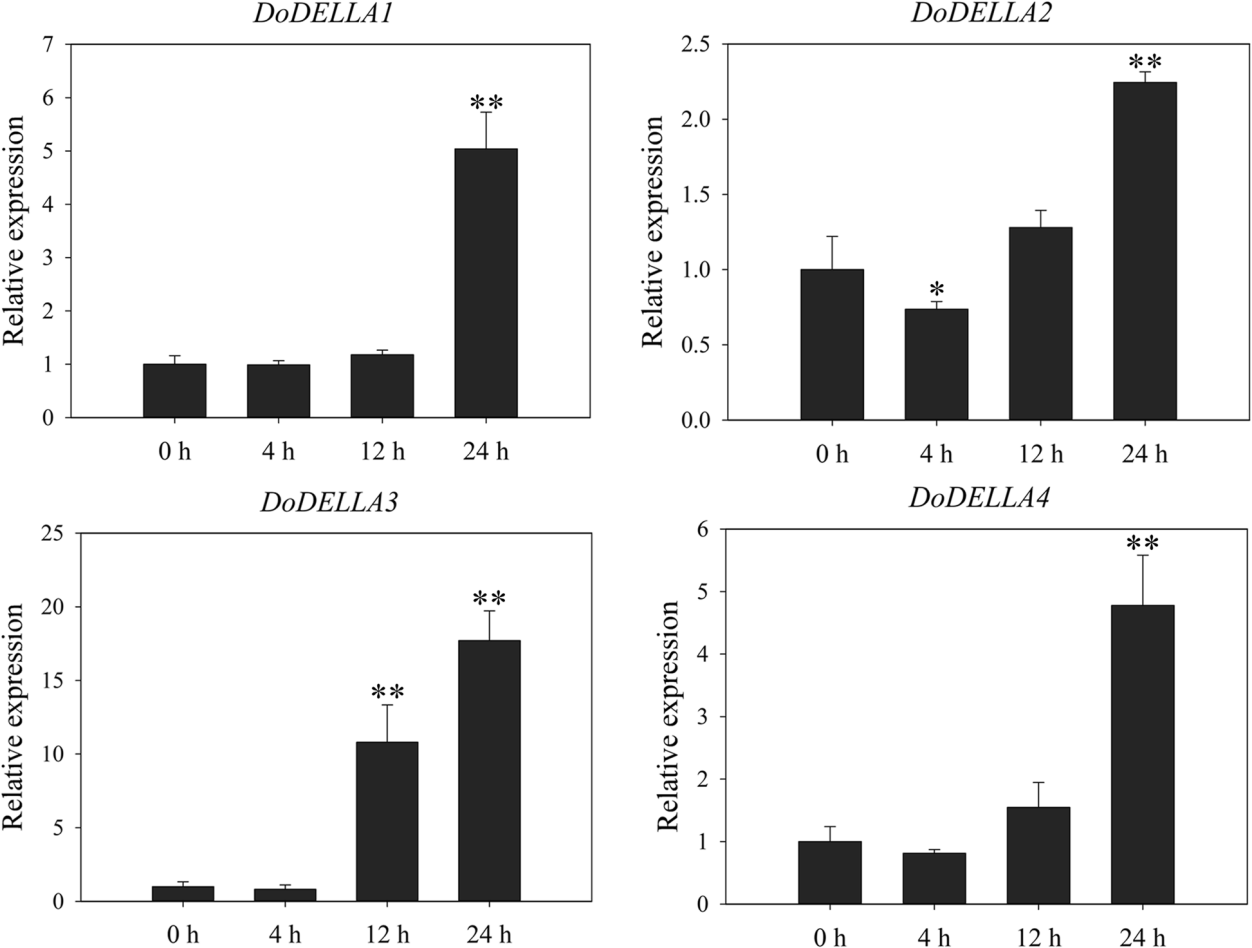
qRT-PCR-based expression analysis of four *DoDELLA* genes in the leaves of *Dendrobium officinale* in response to 100 μm GA_3_. Data indicates the mean ± SD of three biological replicates (*n* = 3). Asterisks denote statistically significant differences: * *p* < 0.05; ** *p* < 0.01

**Fig. 5:**
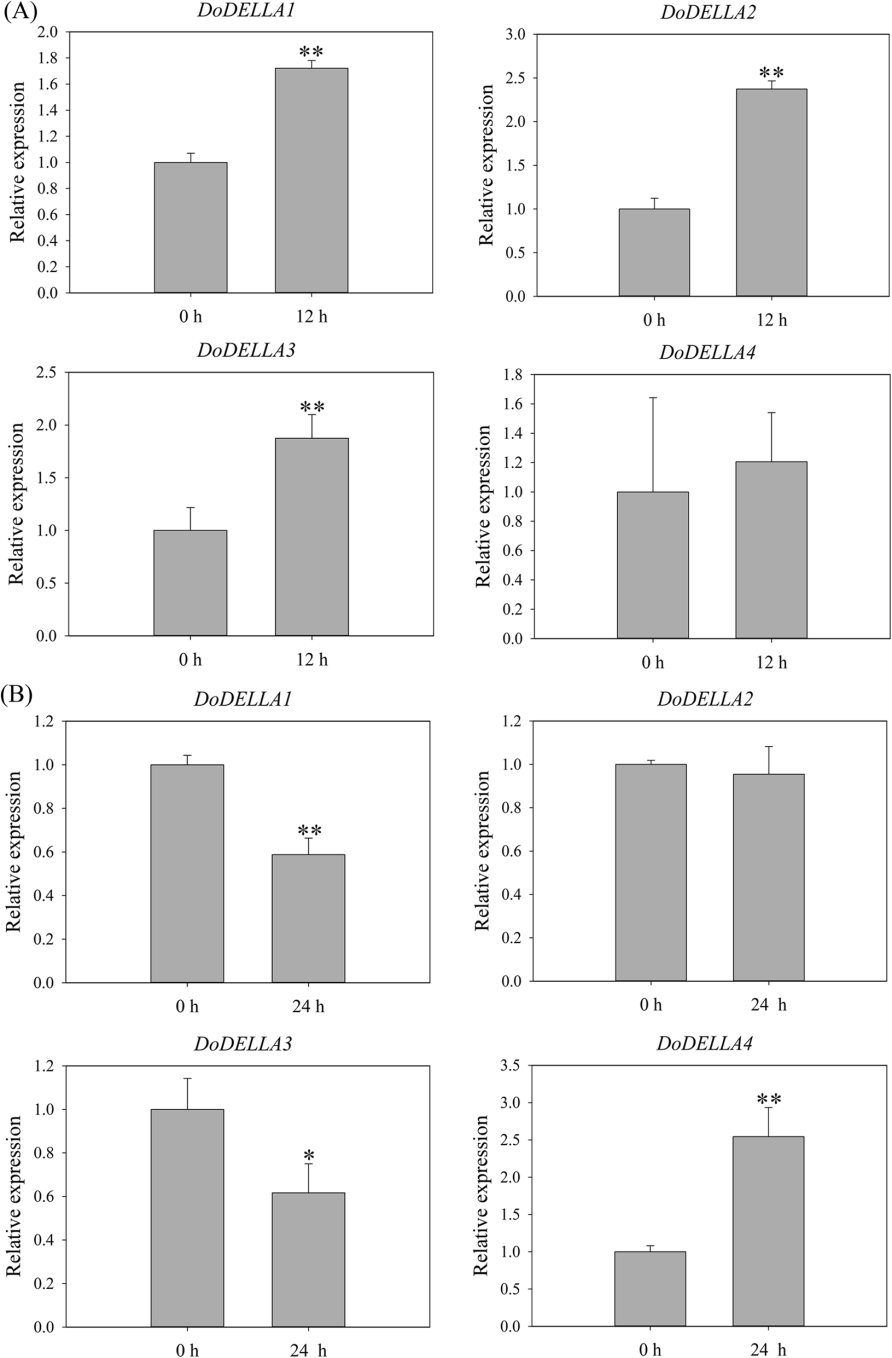
qRT-PCR-based expression analysis of *DoDELLA* genes in roots of *Dendrobium officinale* plantlets exposed to two abiotic stress treatments. **A** NaCl (250 mM) treatment; **B** Drought (15% PEG 6000) treatment. Data indicates the mean ± SD of three biological replicates (*n* = 3). Asterisks denote statistically significant differences: * *p* < 0.05; ** *p* < 0.01

DELLA proteins mediate GA signaling [45]. Quantitative real-time polymerase chain reaction (qRT-PCR) was used to assess the expression patterns of the four *DoDELLA* genes in response to GA_3_ (Fig. 4) and two abiotic stresses (NaCl and polyethylene glycol (PEG)) (Fig. 5). The roots, stems and leaves of *D. officinale* seedlings treated with GA_3_, NaCl and PEG at different times were used as the experimental group while untreated seedlings served as the control group (0 h, not sprayed with GA_3_, NaCl or PEG solutions).

In the GA_3_ treatment, and compared to the control (no GA_3_), the expression of all four *DoDELLA* genes increased significantly after 24 h, and *DoDELLA1* and *3* were up-regulated 5.04- and 17.71-fold, respectively (Fig. 4). Compared with the control treatment, the expression of *DoDELLA* genes in leaves in the GA_3_ treatment was larger than in roots and stems (Fig. S1). These results suggest that *DoDELLA* genes are induced by GA_3_, with enhanced expression in leaves, implying their involvement in the GA regulatory network.

*DELLA* genes respond to abiotic stresses [46]. The expression levels of the four *DoDELLA* genes in roots were similar under salt stress but were different under drought stress (Fig. 5). Under salt stress, the expression of *DoDELLA1-4* was up-regulated after 12 h, *DoDELLA1-3* being very highly expressed, with *DoDELLA1* expression being 1.72-fold higher than the control (Fig. 5A). Surprisingly, exposure to 15% PEG 6000 treatment (i.e., a drought stress) for 24 h resulted in the 1.70- and 1.62-fold down-regulation in the expression of *DoDELLA1* and *3*, respectively (Fig. 5B). These results suggest that both *DoDELLA1* and *3* respond to abiotic (salt and drought) stresses, although the response mechanism to each stress differs. *DoDELLA1* and *3* may play a role in abiotic stress resistance. In addition, the expression of *DoDELLA1-4* differed depending on the organ (roots, stems, and leaves) (Figs. 5, S2 and S3), suggesting a developmental stage-specific nature of their expression (up- or down-regulation).

### Subcellular localization of DoDELLA1 protein

**Fig. 6:**
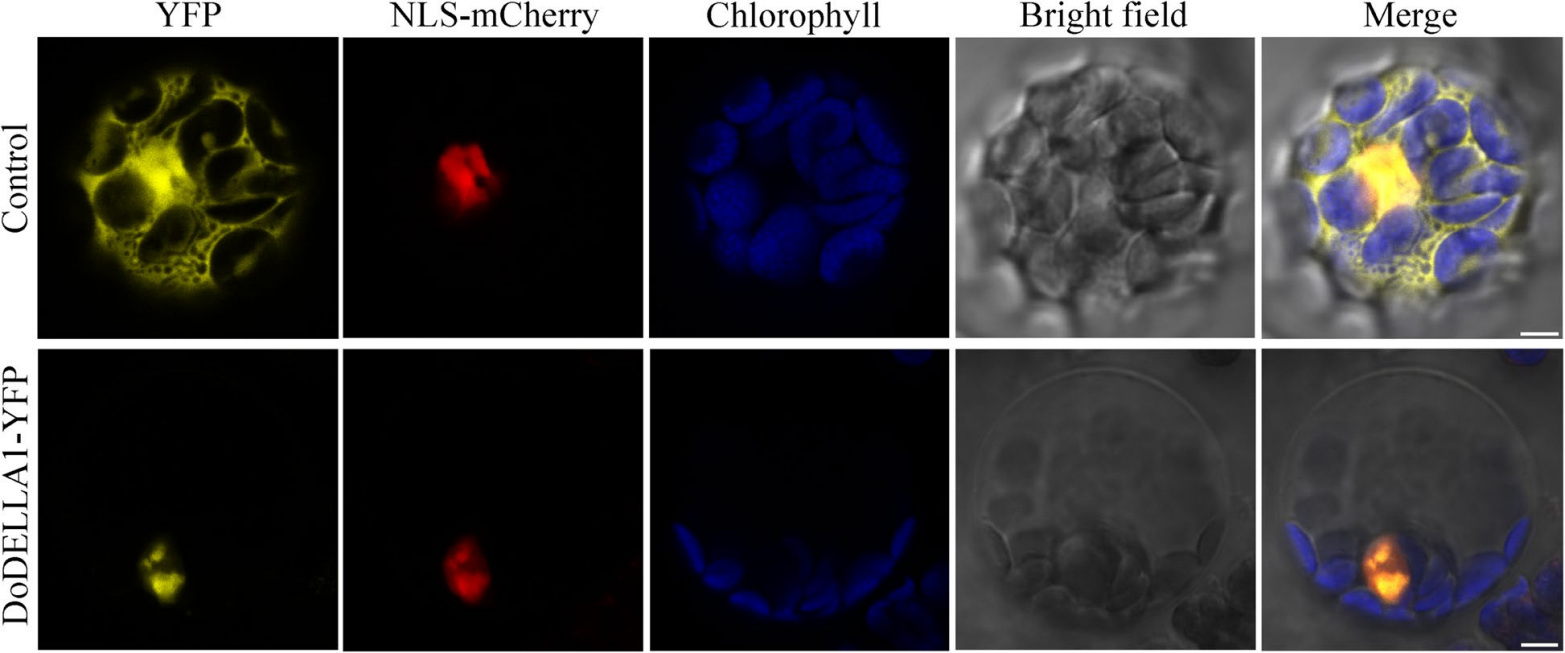
Subcellular localization of control YFP and DoDELLA1-YFP with the nucleus localization marker NLS-mCherry in *Arabidopsis thaliana* mesophyll protoplasts. The control YFP was co-transfected with empty YFP and NLS-mCherry plasmids. Bars = 5 μm

DELLA protein is located in the nucleus, which is consistent with the role of regulation of its gene expression [16, 47]. To further analyze the function of DoDELLA proteins, we selected DoDELLA1 as a representative for further analysis (see the discussion for reasons for this selection). For the subcellular localization analysis, the positive controls (empty yellow fluorescent protein (YFP) and nuclear localization sequence (NLS)-mCherry plasmids) showed strong yellow fluorescence (by YFP) signals in the cytoplasm and plasma membrane (Fig. 6), while YFP fluorescence of the YFP-DELLA1-fused protein was located in the nucleus (Fig. 6), implying that DELLA1 is localized in the nucleus where it may function as a TF.

### Transcriptional activity of DoDELLA1 protein in yeast

DELLA family members are TFs so they possess defining features of TFs like transactivation activity. Transcriptional activity of DoDELLA1 was investigated using a yeast-based system to identify the corresponding characteristics of this DoDELLA protein.

**Fig. 7:**
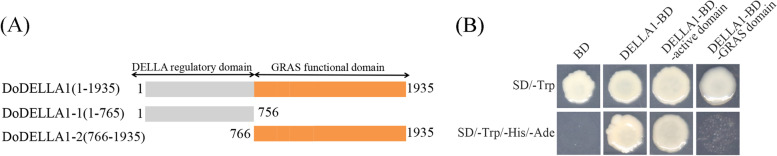
Transcriptional activation analysis of fragments of the DoDELLA1 protein. **A** Schematic showing the DoDELLA1 deletion constructs tested in the transcriptional activation analysis. The DoDELLA1 protein contains the N-terminal DELLA regulatory domain (1–756 aa) and the C-terminal GRAS functional domain (766–1935 aa). **B** Transcriptional activity analysis of the full length of DoDELLA1, the DoDELLA1-active domain and the DoDELLA1-GRAS domain in yeast. BD: pGBKT7 (a yeast two-hybrid prey expression vector) was used as the negative control; DELLA1-BD: DoDELLA1-pGBKT7; DELLA1-BD-active domain: DoDELLA1-active domain-pGBKT7; DELLA1-BD-GRAS domain: DoDELLA1-GRAS domain-pGBKT7. SD/-Trp: yeast culture medium without tryptophan; SD/-Trp/-His/-Ade: yeast culture medium without tryptophan, histidine and adenine

We inserted the full-length Coding sequence (CDS) of the *DoDELLA1* gene into the pGBKT7 yeast expression vector, which expressed the GAL4 DNA-binding domain (Fig. 7). The empty vector pGBKT7 was used as the negative control. These constructs were transformed into yeast strain AH109. The results indicate that while the negative control pGBKT7 was able to grow in SD/-Trp, it was unable to grow in SD/-Trp/-His/-Ade selection medium (Fig. 7). In contrast, yeast strain AH109, which contained the DoDELLA1 fusion protein cloned into the pGBKT7 vector, grew well in SD/-Trp/-His/-Ade selection medium (Fig. 7). This implies that DoDELLA1 activated transcription in yeast.

The two DNA deletion mutants that were used had a DELLA/TVHYNP motif at the N-terminal DELLA regulatory domain (which possesses transactivation activity, namely the DELLA1-active domain), and the GRAS functional domain at the C-terminal (namely the GRAS domain) [20]. These deletion mutants were combined and cloned into the pGBKT7 vector, transformed into yeast strain AH109, and grown on SD/-Trp plates. The DoDELLA1 protein contains the DoDELLA1-active domain (amino acids 1–765), namely DoDELLA1-1, and the DoDELLA1-GRAS domain (amino acids 766–1935), namely DoDELLA1-2 (Fig. 7A). The yeast strain AH109 containing fragments of the DoDELLA1-active domain could grow in SD/-Trp/-His/-Ade medium whereas the strain containing the DoDELLA1-GRAS domain could not (Fig. 7B). This suggests that the N-terminal DELLA1-active domain has strong self-transactivation activity in yeast whereas the C-terminal GRAS domain exhibited no transcriptional activation activity. These results also suggest that DoDELLA1 may be a transcriptional activator.

### Identification of DoDELLA1-interacting proteins by yeast two-hybrid (Y2H) screening

**Fig. 8:**
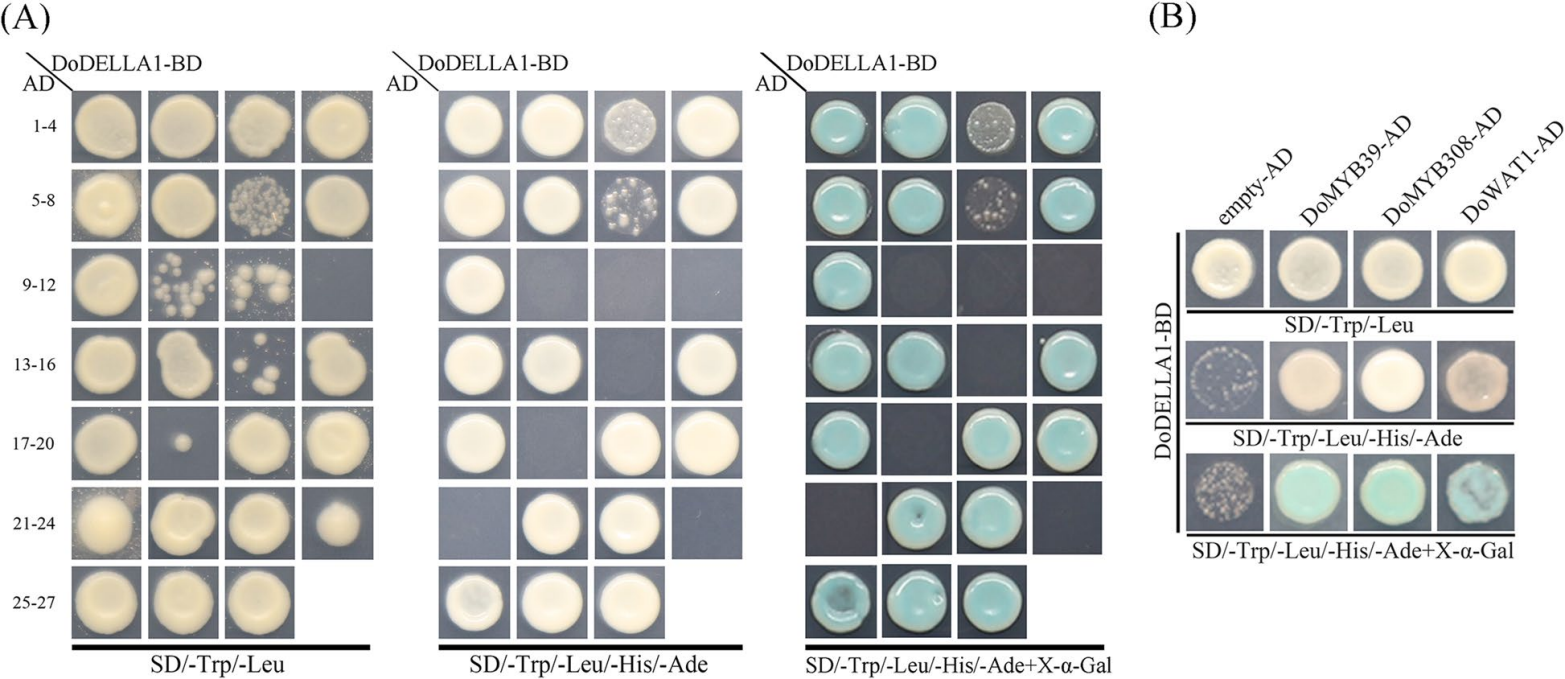
DoDELLA1 yeast two-hybrid (Y2H) library screening assay. The N-terminus of the GRAS domain of DoDELLA1 with a binding function was used for Y2H screening. **A** Y2H screening and positive clone identification. Preliminary screening of 27 transformant colonies on SD medium without Trp and Leu. An additional 18 positive clones were obtained from the interaction between DoDELLA1 and other proteins in an in vitro X-α-gal activity assay, detected on SD medium without Trp, Leu, His and Ade. **B** Analysis of the interaction between DoDELLA1 and DoMYB39, DoDELLA1 and DoMYB308, and DoDELLA1 and DoWAT1, using a Y2H assay. All 18 positive clones were used for comparing sequences, and three key proteins (DoMYB39, DoMYB308 and DoWAT1) were used for Y2H rotary validation. An empty vector was used as the control. AD: pGADT7, a Y2H bait expression vector; BD: pGBKT7, a Y2H prey expression vector; empty, a negative control. SD/-Trp/-Leu: yeast culture medium without tryptophan and leucine; SD/-Trp/-Leu/-His/-Ade: yeast culture medium without tryptophan, leucine, histidine, and adenine; SD/-Trp/-Leu/-His/-Ade + X-α-Gal: yeast culture medium without tryptophan, leucine, histidine, and adenine, to which X-α-Gal was added

Numerous studies have shown that the GRAS domain is crucial to the binding of DELLA family members to other proteins [48]. For example, one region of the GRAS domain, the LHR1 domain, combines with PHYTOCHROME-INTERACTING FACTOR 4 (PIF4) [35, 49], or the jasmonate ZIM domain 1 (JAZ1) [45, 50]. In addition, an N-terminal with strong self-transactivation activity makes Y2H screening more difficult and complex. Therefore, we used a truncated version of DoDELLA1 in which the DELLA1-GRAS domain (amino acids 766–1935) lacked the N-terminal region (amino acids 1–765), thereby removing its self-transactivating activity, as a bait against a TF library for Y2H screening to explore the protein–protein interaction network of DELLA1. This approach not only ensures the detection of fewer false-positive clones, but also preserves the ability to identify the genuine partners of DELLA1. We initially obtained 27 positive clones (Fig. 8A). After further screening *ADE2*, *HIS3* and *LacZ* reporter genes, we obtained 18 positive clones (Fig. 8A). Sequencing results revealed that a total of 14 effectively screened proteins after eliminating duplicates (Table 1). Three key proteins (two TFs i.e., DoMYB39 and DoMYB308, and the DoWAT1 protein) were selected for yeast rotation validation. The results of the transition verification assay of these three proteins showed that they all activated *ADE2*, *HIS3* and *LacZ* reporter genes (Fig. 8B), indicating that they may be reciprocal proteins of DoDELLA1.

**Table 1:** The basic description of DoDELLA1 protein obtained by Y2H hybrid library screening and further sequencing. The sample name in the table were self-named according to the order of positive colonies obtained by yeast two-hybrid library screening and PCR verification

**Sample name**	**Locus**	Accession number	**Description**
(3)	MA16_Dca000522	KZ502442	[product = Tropinone reductase like] [gbkey = mRNA]
(5)	MA16_Dca021117	KZ503199	[product = 3-ketoacyl-CoA synthase 10] [gbkey = mRNA]
(10)	MA16_Dca024031	KZ502068	[gene = MYB308] [product = Myb-related protein 308]
(11)	MA16_Dca018701	KZ502223	[gene = MYB39] [product = Transcription factor MYB39]
(12)	MA16_Dca019300	KZ503487	[product = WAT1-related protein]
(13)	MA16_Dca023783	KZ502224	[product = 21 kDa protein]
(15)	MA16_Dca009670	KZ503270	[product = 60S ribosomal protein L15]
(16)	MA16_Dca005564	KZ502486	[product = ribonuclease III family protein]
(18)	MA16_Dca006644	KZ502134	[product = hypothetical protein]
(19)	MA16_Dca018511	KZ503951	[gene = LHCA4][product = Chlorophyll a-b binding protein 4, chloroplastic]
(21)	MA16_Dca001054	KZ502537	[product = Defensin-like protein]
(22)	MA16_Dca006914	KZ503267	[gene = UBC2] [product = Ubiquitin-conjugating enzyme E2 2]
(23)	MA16_Dca025851	KZ503804	[gene = TIF3A1] [product = Eukaryotic translation initiation factor 3 subunit A]
(27)	MA16_Dca023835	KZ502566	[gene = IN2-2] [product = IN2-2 protein]

## Discussion

### The DELLA gene family in *D. officinale*

Development in *D. officinale* is a complex set of processes that are regulated by numerous genes and phytohormones. The number of DELLA gene family members is small, and they are plant-specific TFs that belong to a subgroup of the GRAS family [51, 52]. For example, five DELLA proteins were identified in *A. thaliana* [53] or only a single DELLA protein (SLR1) in rice [17]. In this study, four DoDELLLA TFs were identified in *D. officinale* based on a neighbor-joining (NJ)-based method, which indicated that *D. officinale* had several genes that were homologous to rice *SLR1*. In contrast, these *D. officinale* DELLA proteins could not be divided into different clades based on the classification of *Arabidopsis* DELLA proteins. Based on sequence similarity, the GAI and RGA clade belonged to group I, the RGL1 clade belonged to group II, and the RGL2 and RGL3 clade belonged to group III [54], so they displayed large evolutionary distances (Fig. 1). The four DoDELLA proteins might thus need to be categorized as a separate clade. Unfortunately, since DELLA proteins are rarely reported and are still poorly understood in orchids, no robust reference currently exists for the classification of DoDELLA proteins. The DELLA protein has been characterized as a kind of evolutionarily conserved protein [5]. The genes encoding DELLA proteins have been identified in a variety of plants, but most are model plants or crops such as *A. thaliana* [9, 16, 24], maize [15], wheat [15], rice [17], and barley [55], in which DELLA proteins were studied to improve harvest index, fruit quality, tillering, stress tolerance, and regulation of flowering time. In this study, the four DoDELLA proteins were genetically close to rice, and the DELLA proteins of monocotyledonous plants (*O. sativa* and *D. officinale*) and a dicotyledonous plant (*A. thaliana*) were clustered into two distinct clades (Fig. 1). This is consistent with their evolutionary pattern in nature, and further implies that DELLA proteins may be closely related to differentiation-related processes in these two classes of plants. Some studies found that DELLA members belonging to the same subfamily had overlapping or diverse functions [24, 56]. It is unclear why rice, which has a larger genome than *A. thalia*na [57], has fewer DELLA proteins. A previous report on gene family contraction events showed a possible association with genetic loss [58], perhaps affecting the number of DELLA proteins in evolution. Separately, studies showed that even though one protein in *Picea abies* displayed > 80% sequence similarity with a *Pinus pinaster* DELLA protein, it was not considered to be a DELLA gene because it did not contain a DELLA domain, so perhaps it can be inferred that this is a DELLA gene loss event in *Picea abies* [59].

### *DoDELLA* genes play important roles in shoot regeneration

The DELLA protein, which is a well-characterized nuclear-localized TF that is involved in a range of plant developmental processes, responds to abiotic and biotic stresses, and can be modulated by crosstalk between GA and various hormone signals [34, 60, 61]. In addition to the large aforementioned differences in the number of DELLA gene family members between plant species, the expression levels of DELLA genes can also vary considerably in different tissues of the same species. For example, in *A. thaliana*, AtGAI and AtRGA were expressed in all tissues, while three additional DELLA proteins, AtRGL1, AtRGL2 and AtGRL3, were expressed only in flowers, seeds and fruits [22]. In this study, the expression of all *DoDELLA* genes was highest in MSs (Fig. 3), suggesting their importance in regulating shoot regeneration in *D. officinale*. This is consistent with the involvement of DELLA in the regulation of shoot regeneration in tomato [62]. In general, MSs are produced by differentiating adventitious shoots from enlarged apical meristematic tissues [63]. This is a key stage that ensures development and regeneration in this orchid. The expression pattern of a gene is often related to its function [64]. Early studies on MSs in maize showed that vegetative shoot tips effectively produced MSs through adventitious bud formation and somatic embryogenesis, and transgenic maize lines were successfully obtained by genetic transformation of MSs using microprojectile bombardment [65, 66]. Additionally, successful transgenesis using MSs generated by micropropagation has been achieved in orchid genera such as *Dendrobium*, *Paphiopedilum* and *Orchis* [67–69]. This provides a possibility for the breeding of transgenic orchids using MSs. *D. officinale* is traditionally propagated by seed germination [70], but this method is time-consuming and is limited by low germination rate, shallow rooting and the lack of high-quality planting materials [71, 72]. All four *DoDELLA* genes were highly expressed at this specific developmental stage, i.e., MSs, indicating that they may have an important regulatory role in the regeneration of MSs and shoots. Even though plant regeneration is now widely used in orchid biotechnology [67, 68], the key regulatory networks in orchids remain largely unknown, and there are few studies on DELLA regulating shoot regeneration. Therefore, further analysis of the mechanism of shoot regeneration regulated by DoDELLAs has a broad application market in *D. officinale* breeding.

### *DoDELLA* genes respond to exogenously-applied GA_3_

Plant growth and development are affected by the interaction of various hormones. All *DoDELLA* genes were induced by GA_3_ treatment and analysis of their expression profiles 24 h after GA_3_ treatment showed a consistent trend of significantly increased expression (Fig. 4). A phylogenetic analysis clustered DoDELLA1 and 2 into one group (Fig. 1), implying that DoDELLA1 may have a similar function as DoDELLA2. Of particular note are DoDELLA1 and 3, which were highly up-regulated in leaves in response to GA_3_ treatment, suggesting that they may be GA-responsive genes with a potential role in GA regulation during the development of leaves. GA and abscisic acid (ABA) play antagonistic roles in regulating seed germination. In *A. thaliana*, DELLA proteins interact with regulators of the ABA signaling pathway, affecting ABA biosynthesis and inhibiting GA biosynthesis, ultimately inhibiting seed germination [73, 74]. In rice, GA is pivotal for rice flower development [75], and the DELLA protein SLR1 and flowering-related protein CKI together negatively regulate GA signaling [76]. In addition to GA, DELLA protein also plays an important role in signal transduction of other plant hormones. In *A. thaliana*, ethylene inhibits root elongation by delaying GA-induced degradation of DELLA proteins [77]. During the development of tomato fruit, the auxin signal component SIARF7, activator SiARFs and the GA signal inhibitor SiDELLA co-regulate the expression of fruit growth-related genes [78]. Jasmonic acid (JA) and AtDELLA proteins interact to promote stamen development, and its binding to JAZ1 also affects the accumulation of JA [50, 79]. The precise mechanism by which DELLA proteins integrate in hormone signal transduction is unknown, and may be a research hotspot in the future.

### Regulatory and interaction networks among DELLA proteins and other TFs

DELLA protein can combine with a variety of proteins to form protein complexes in order to work together [80]. DELLA protein can be specially combined with the downstream target gene of the GA signal channel, regulating the expression of downstream genes, then regulating plant growth and development [36]. DELLA proteins, acting as co-repressors, suppressed gene transcription by interacting with a key flowering protein, FLOWERING LOCUS C (FLC), thereby repressing the transition to flowering in *A. thaliana* [81]. Somewhat surprisingly, the expression patterns of *DoDELLA1-4* were remarkably consistent at three different developmental stages, with their expression levels decreasing sequentially in MSs, plantlets, and PLBs (Fig. 3), suggesting that their functions may overlap or be similar to some extent. Two *A. thaliana* DELLA proteins, GAI and RGA, had an overlapping function in the inhibition of plant stem growth [43, 82]. Similarly, the expression patterns of the WOX TFs, PRESSED FLOWER (PRS) and WOX1, overlapped or were similar [83]. These lines of evidence indicate that different DELLA TFs can share some common response mechanisms in regulating plant growth and development. The phylogenetic tree-based genetic distance of DoDELLA1 and 2 was closest to the DELLA proteins of *O. sativa* and *A. thaliana* (Fig. 1). DoDELLA1 and 3 showed a large increase in expression in response to exogenous GA_3_ in *D. officinale* leaves (Fig. 4). For these reasons, we analyzed DoDELLA1 in detail via functional analysis.

**Fig. 9:**
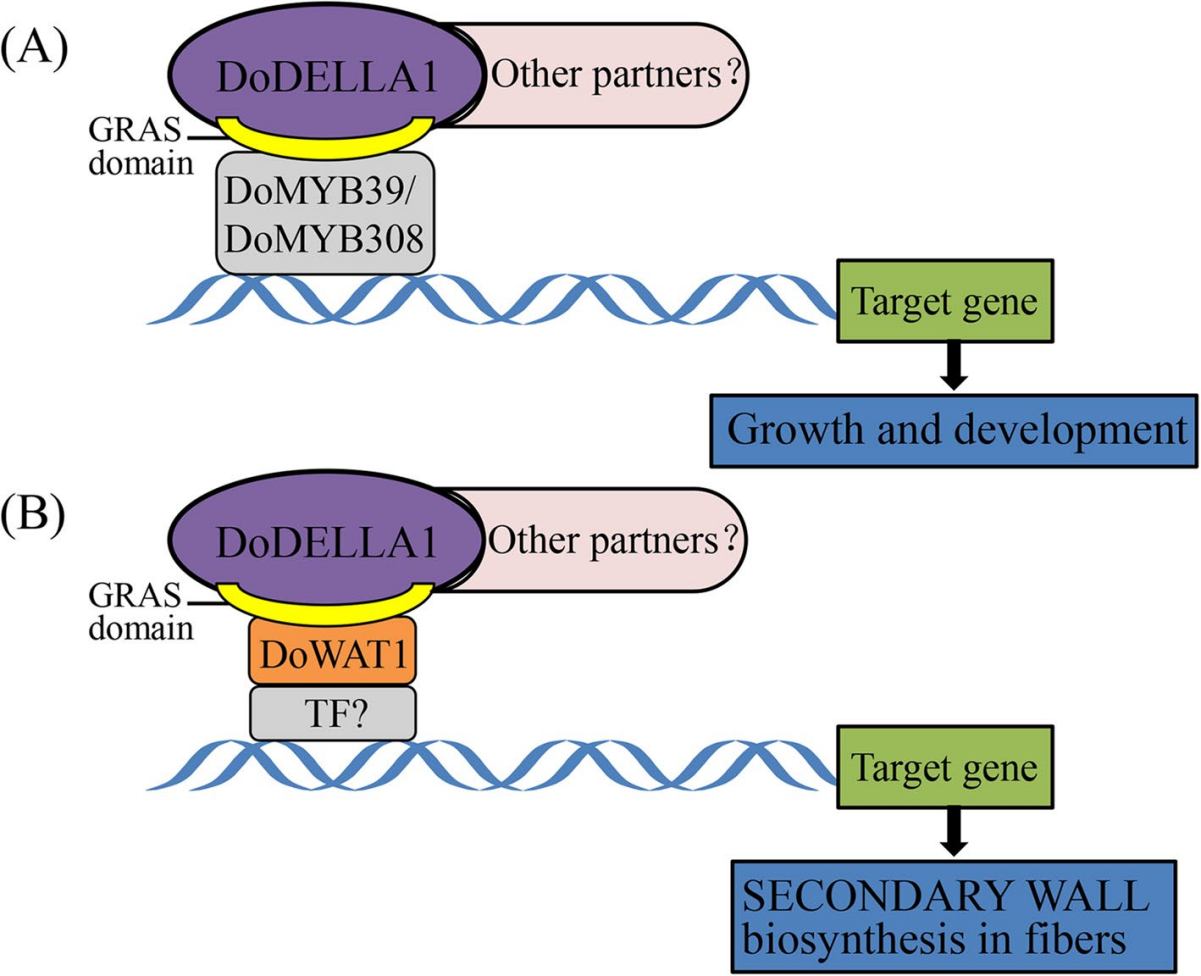
A simple model depicting how DoDELLA1 might interact with transcription factors (TFs) (e.g. DoMYB39 and DoMYB308) or other proteins (e.g. DoWAT1) to form complexes to activate the expression of downstream target genes, thereby regulating the development of *D. officinale*. **A** The GRAS domain of DoDELLA1 interacts with the TFs DoMYB39 or DoMYB308 to regulate growth and development. **B** The GRAS domain of DoDELLA1 interacts with DoWAT1 protein to regulate SECONDARY WALL biosynthesis in fibers

To facilitate Y2H screening, DELLA1 was truncated into two segments [17, 24, 84], one near the N-terminal DELLA regulatory domain to isolate the DELLA/TVHYNP motif and the other near the C-terminal to isolate the GRAS domain. DoDELLA1 displayed transcriptional activity (Fig. 7). Truncated DoDELLA1, after removing the activated domain, included the GRAS domain with a binding function, which is consistent with what was observed in previous studies [26, 42, 52]. Furthermore, we screened the interaction of truncated DoDELLA1 with DoMYB39 and DoMYB308 TFs, or with DoWAT1, using Y2H screening (Fig. 8B). The MYB gene family is widely involved in plant growth and development, not only in model plants, but also in major crops and horticultural plants [85–88]. Studies on MYB in *D. officinale* mainly focused on polysaccharide synthesis [89], flower development [89], stress response [90, 91], alkaloid biosynthesis [92] and terpenoid biosynthesis [93], suggesting that DoDELLA1 and DoMYBs may co-regulate one or more aspects of *D. officinale*, although the related functions need to be further explored and verified. WALLS ARE THIN1 (WAT1) is a plant-specific protein that regulates the secondary cell wall thickness of stem fibers and participates in the integration of auxin signaling in *A. thaliana* fibers [94, 95]. In both poplar and the *A. thaliana* mutant *wat1*, auxin content decreased significantly [94, 96]. Since WAT is closely related to DELLA in poplar [96], this partially supports the idea that DoDELLA1 may interact with DoWAT1 in this study (Fig. 8B). Moreover, SLWAT1 was activated by brassinosteroid (BR) i.e., a BR-activated WAT1 module to enhance auxin signaling, facilitating wood formation in tomato (*Solanum lycopersicum* L.) [97]. Subsequently, secondary WALL ASSOCIATED NAC DOMAIN PROTEIN 1 (SND1) was involved in the regulation of SECONDARY WALL biosynthesis in fibers [98], and several TFs such as MYB46, MYB103, and KNAT7 are direct targets of SND1 [99, 100], suggesting that DoMYB proteins and DoWAT1 may lie within the same regulatory pathway in *D. officinale*. Plant DELLA proteins, in the form of protein complexes, are also widely involved in the regulation of other pathways related to growth and development. For example, the DELLA-TOC159 protein complex interaction in *A. thaliana* controls chloroplast formation during early plant development [101] while the interaction between DELLA and PIF proteins in tomato regulate the balance of plant hormones in vivo [102]. *DoDELLA1-4* displayed tissue-specific expression and played different biological functions in different tissues (Figs. 5, S2 and S3), DELLA protein is thus involved in the morphogenesis of different tissues and organs, GA signal transduction and stress response, but the precise regulation pathway of DELLA protein in the growth and development of *D. officinale* needs to be studied further. DELLA protein can combine with a variety of proteins into a protein complex to play a role. DELLA protein can specifically combine with downstream target genes of GA signaling, regulating their expression and then regulating plant growth and development [36]. Based on the present study, we propose a simple model in which DoDELLA1 might interact with TFs or other proteins to regulate *D. officinale* development (Fig. 9).

## Conclusion

In conclusion, four DoDELLA proteins (DoDELLA1-4) were identified in a popular Chinese herbal orchid, *D. officinale*. The expression patterns of the four *DoDELLA* genes at different developmental stages, organs, stress treatments, and in response to exogenously applied GA_3_, were analyzed. Furthermore, the localization and transcriptional activity of DoDELLA1 were evaluated. Three DoDELLA-protein interaction pairs (DoDELLA1-DoMYB36, DoDELLA1-DoMYB308 and DoDELLA1-DoWAT1) were screened by Y2H screening, laying a foundation for further analysis and functional verification. Our results provide a basis to explain the specific expression and regulation of different developmental stages and tissues in *D. officinale*. Our findings contribute key information for elucidating signaling events downstream of *DoDELLA* to understand how GA controls plant development. This study helps to understand the functions of DELLA genes in orchids, providing clues for exploring the regulatory networks that control growth and stress responses, eventually allowing the establishment of breeding programs that employ transgenic techniques to improve growth, development, productivity, and biotic or abiotic stress resistance.

## Materials and methods

### Plant materials and growth conditions

This study used *D. officinale* plants that were grown and maintained in the South China Botanical Garden, Chinese Academy of Sciences, in Guangzhou, China. PLBs, MSs (i.e., shoots without roots) and plantlets (about 5 cm tall) were grown on half-strength Murashige and Skoog (½MS) [103] medium to which 20 g/L sucrose, 6 g/L agar and 1 g/L activated charcoal (pH 5.4) were added. Cultures were maintained in a growth chamber. According to a previous study [104], *D. officinale* seedlings (about 8–9 cm in height) were sprayed with one of three abiotic stressors: 100 μM GA_3_ (Yuanye Biotechnology Co., Ltd., Shanghai, China) for 0, 4, 12 and 24 h, 250 mM NaCl (Guangzhou Chemical Reagent Factory, Guangzhou, China) for 0 and 12 h, and 15% PEG 6000 (Solarbio Science & Technology Co., Ltd., Beijing, China) for 0 and 24 h. Greenhouse plants were grown at 75–80% relative humidity, 28/25 °C (day/night mean temperatures), and a 12-h photoperiod. The ambient conditions of the growth chamber with in vitro cultures were about 60% relative humidity, 26 ± 1℃, a photosynthetic photon flux density of 86.86 μmol·m^−2^·s^−1^, and a 12-h photoperiod. Each treatment was conducted as three replications. Samples (i.e. PLBs, MSs, plantlets; roots, stems and leaves were sampled from *D. officinale* seedlings) from any treatment were immediately frozen in liquid nitrogen for 15 min then stored at -80℃ for later use.

### Identification of *DoDELLA* genes in the *D. officinale* genome

The National Center for Biotechnology Information (NCBI, https://ftp.ncbi.nlm.nih.gov) was assessed to obtain *D. officinale* protein sequences. At first, the sequences of five *A. thaliana* DELLA proteins (GA-INSENSITIVE (GAI), REPRESSOR OF GAl-3 (RGA), and RGA-LIKE1, i.e., RGL1, RGL2 and RGL3) were downloaded from the TAIR database (http://www.arabidopsis.org/). HMMER 3.0 software [105] was then used to assess the hidden Markov model (HMM) profile under default parameters (http://hmmer.janelia.org/). Based on the HMM profile of the DELLA domain, we identified all possible DELLA protein candidates in *D. officinale*. Online software SMART (http://smart.embl-heidelberg.de/) [106] was used to integrate DELLA domains for the identification of the *DELLA* gene family in *D. officinale*. Sequences with 80% similarity were selected, and ScanProsite (https://prosite.expasy.org) [107] and InterProScan (https://www.ebi.ac.uk/interpro/search/sequence/) [108] were used to manually re-annotate the DELLA proteins containing incorrect or multiple termination signals or repeated sequences. Finally, four genes (*DELLA1*, *2*, *3* and *4*) were identified. These were considered to be *D. officinale DELLA* (i.e., *DoDELLA*) genes.

### Bioinformatics analysis of DoDELLA proteins

Multiple sequence alignments of full-length amino acid sequences of the four DoDELLA proteins were generated using DNAMAN version 8.0 software (Lynnon Biosoft, Foster City, CA, USA). Using Clustal X version 2.0 [109], the DoDELLA protein sequences were then aligned to further verify the results of sequence alignment. Based on the alignment of DELLA proteins, an evolutionary tree was constructed with MEGA version 7 software [110] using the NJ method [111]. The NJ tree was constructed with the Phylogeny Inference Package (PHYLIP) software package version 3.2 [112]. The authenticity of the tree was tested by performing 1000 bootstrap replications to finally construct a phylogenetic tree of *D. officinale*, *O. sativa* and *A. thaliana* DELLA proteins. In order to classify their characteristic domains, the full-length amino acid sequences of the four DoDELLA proteins were aligned in DNAMAN version 8.0 software (Lynnon Biosoft) using default parameters.

### RNA extraction, cDNA synthesis and qRT-PCR

Based on the manufacturer’s instructions, the Quick RNA extraction kit (Huayueyang Biotechnology Co. Ltd., Beijing, China) was used to extract total RNA from each sample (i.e., the aforementioned *D. officinale* materials from different treatments of three stresses) (0.5 g, three independent biological replicates). RNA that was obtained was purified with RNase-free DNase I (Takara Bio Inc., Kyoto, Japan) and after running 2 μL of purified RNA by agarose gel electrophoresis, its integrity was checked with the Clinx GenoSens gel documentation system (Clinx Science Instruments, Shanghai, China). RNA concentration and quality were assessed with a NanoDrop 2000c Spectrophotometer (Thermo Scientific, Wilmington, NC, USA). Following the manufacturer’s protocol, the GoScript™ Reverse Transcription System (Promega, Madison, WI, USA) was used to reverse transcribe 4 g of purified RNA. cDNA was diluted with ddH_2_O at a ratio of 1:10 (w/v) and used as a template for qRT-PCR analysis. The *D. officinale ACTIN* gene (NCBI accession number: JX294908) was used as the internal reference gene [113] to normalize cDNA concentration. qRT-PCR was performed using the Aptamer™ qPCR SYBR® Green Master Mix (Tianjin Novogene Bioinformatics Technology Co. Ltd., Tianjin, China) in the LightCycler 480 system (Roche, Basel, Switzerland) at an established set of reaction conditions (95℃ for 5 min; 40 cycles of 95℃ for 10 s; 60℃ for 1 min). The 2^−ΔΔCT^ method [114] was used to calculate relative gene expression. Supplementary Table S1 lists the primer sequences for all *DoDELLA* genes analyzed by qRT-PCR.

### Subcellular location analysis of DoDELLA1 protein

The *DoDELLA1* gene fragment without a stop codon was amplified by PCR then inserted into the *Eco*RI site of the pSAT6-EYFP-N1 expression vector to construct a YFP-fusion vector [115]. *A. thaliana* mesophyll protoplasts, which are useful for subcellular localization experiments, were used as the transient gene expression system. Protoplasts were isolated from the mesophyll of leaves of four-week-old *A. thaliana* plants [116]*.* Using a PEG-mediated method [116], recombinant plasmids (YFP-DoDELLA1) and NLS localization marker (NLS-mCherry) were co-transformed into protoplasts. A Leica TCS SP8 STED 3 × microscope (Leica Microsystems, Wetzlar, Germany) was used to detect YFP and NLS fluorescence signals in protoplasts after incubation in the dark for 12–18 h. Supplementary Table S2 lists the primer pairs that were used to generate the YFP-DoDELLA1 fusion protein.

### Transactivation activity analysis and the screening of two-hybrid yeast libraries

The cDNA libraries were used for the Y2H library screening assay by following the manufacturer’s instructions (Shanghai Hitech Bio-Technology Co. Ltd., Shanghai, China). Since the DELLA1 protein contains a GRAS domain and an active domain, it was divided it into a full-length DELLA1 protein (i.e., DELLA1-GRAS) and two truncated DELLA1 proteins (i.e., DELLLA1-active1 and DELLLA1-active2). The amplified full-length DELLA1 and truncated DELLA1 were separately connected to the vector pGBKT7 (Clontech, Palo Alto, CA, USA) by homologous recombination to obtain the pGBKT7-DoDELLA1 and truncated pGBKT7-*DoDELLA1*-*X* (pGBKT7-DoDELLA1-GRAS and pGBKT7-DoDELLA1-active) vectors. A transactivation activity detection experiment was performed on DoDELLA1, including the full-length and truncated forms. The three construction vectors (pGBKT7-DoDELLA1, pGBKT7-DoDELLA1-GRAS and pGBKT7-DoDELLA1-active), as well as the negative control (empty vector pGBKT7), were separately transformed into yeast (*Saccharomyces cerevisiae*) strain AH109 according to the manufacturer’s instructions (Weidi Biotechnology Co., Shanghai, China). They were sequentially plated on dropout nutrient medium i.e., SD/-Trp, SD/-Trp/-His solid media plates containing 5-bromo-4-chloro-3-indolyl-α-D-galactoside (X-α-Gal) (Coolaber, Beijing, China) to test for α-galactosidase (MEL1) activity by incubating plates upside down for 3 days in a 29 °C incubator to evaluate whether the transcriptional activity of the full-length pGBKT7-DoDELLA1 and truncated pGBKT7-*DoDELLA1-X*.

In addition, truncated DELLA1-GRAS protein without transcriptional activity was selected for further Y2H screening. Bait plasmid pGBKT7-DELLA1 GRAS protein and the negative control (empty vector pGBKT7) were co-transformed into competent yeast AH109. Following guidance provided by the manufacturer’s instructions, the obtained yeast transformant was used as the recipient strain to prepare competent colonies carrying the library plasmid pGADT7-NNJ-cDNA (Shanghai Hitech Bio-Technology Co. Ltd.). Yeast colonies were spread on SD/-Trp/-Leu/-His solid media plates (Coolaber) and incubated at a constant temperature (30 °C) for 3 days. A total of 27 single colonies of positive clones were picked from the screening library plates and transferred to SD/-Trp/-Leu plates and cultured at constant 30 °C for 2–3 days. The 27 initially positive clone transformants grown on SD/-Trp/-Leu plates were diluted with sterile distilled water, seeded on double synthetic dropout nutrient medium (SD/-Trp/-Leu) and quadruple dropout nutrient medium (SD/-Trp/-Leu/-His/-Ade containing X-α-Gal), then cultured at constant 30 °C for 3–4 days. Resulting positive colonies were detected by PCR and agarose gel electrophoresis, and positive colonies displaying bands after electrophoresis were sent to Sangon Biotech (Shanghai, China) for sequencing. The resulting sequences were compared with sequences from the *D. officinale* genome database [117] to identify the screened positive cloned proteins. Finally, target proteins displaying a potential interaction with DELLA1 were selected. In addition, in order to determine the interaction between the screened protein obtained and DELLA1-GRAS protein in yeast AH109, the full length of the screened protein was cloned by PCR. Subsequently, yeast rotation validation experiments were performed: the obtained candidate protein was transformed into pGADT7 vector (Clontech) then co-transformed into yeast AH109 with pGBKT7-DELLA1-GRAS protein or with the negative control (empty vector pGBKT7), respectively, and the remaining steps were performed as Y2H screening. Supplementary Table S3 lists the specific primer sequences of DoDELLA1, truncated DELLA1 and screened positive proteins used for yeast analysis.

### Statistical analysis

Data were plotted as means ± standard deviation (SD). To detect significant differences (*p* < 0.05 and *p* < 0.01) between treatment means of qRT-PCR experiments, analysis of variance (ANOVA) was followed by Duncan’s multiple range test (DMRT). Analyses were conducted using SPSS version 22.0 software (IBM Corp., Armonk, NY, USA).

## Supplementary Information

**Additional file 1: Figure S1.** Expression analysis of *DoDELLA* genes in leaves of *Dendrobium officinale* roots and stems in response to 100 μm GA_3_ by qRT-PCR. **Figure S2.** Expression analysis of *DoDELLA* genes in stems and leaves under NaCl (250 mM) stress by qRT-PCR. **Figure S3.** Expression analysis of *DoDELLA* genes in stems and leaves) under drought (15% PEG) stress by qRT-PCR.** Table S1.** Primers used for qRT-PCR.** Table S2.** Primers used for subcellular localization analysis.** Table S3.** Primers used for the yeast two-hybrid assay.

## Data Availability

All data generated or analyzed during this study are included in this published article and its supplementary files. All data and plant materials used in current study are available from the corresponding author upon reasonable request.
