# Correction: Uncovering the involvement of DoDELLA1-interacting proteins in development by characterizing the DoDELLA gene family in *Dendrobium officinale*

**DOI:** 10.1186/s12870-023-04184-0

**Published:** 2023-04-01

**Authors:** Danqi Zeng, Can Si, Jaime A. Teixeira da Silva, Hongyu Shi, Jing Chen, Lei Huang, Juan Duan, Chunmei He

**Affiliations:** 1 Key Laboratory of South China Agricultural Plant Molecular Analysis and Genetic Improvement, Provincial Key Laboratory of Applied Botany, South China Botanical Garden, Chinese Academy of Sciences, Guangzhou, 510650 China; 2 South China National Botanical Garden, Guangzhou, 510650 China; 3 University of the Chinese Academy of Sciences, Beijing, 100049 China; 4 Independent Researcher, Ikenobe 3011-2, Miki-cho, Kagawa-Ken, 761-0799 Japan

**Correction:** ***BMC Plant Biol*** **23, 93 (2023)**

**https://doi.org/10.1186/s12870-023-04099-w**

Following publication of the original article [1], an error was identified in the Conclusions section.

The updated conclusion is given below and the changes have been highlighted in **bold typeface**.

## Conclusion

In conclusion, four DoDELLA proteins (DoDELLA1-4) were identified in a popular Chinese herbal orchid, *D.officinale*. The expression patterns of the four *DoDELLA* genes at different developmental stages, organs, stress treatments, and in response to exogenously applied GA3, were analyzed. Furthermore, the localization and transcriptional activity of DoDELLA1 were evaluated. Three DoDELLA-protein interaction pairs (**DoDELLA1-DoMYB39**, DoDELLA1-DoMYB308 and DoDELLA1-DoWAT1) were screened by Y2H screening, laying a foundation for further analysis and functional verification. Our results provide a basis to explain the specific expression and regulation of different developmental stages and tissues in *D. officinale*. Our findings contribute key information for elucidating signaling events downstream of *DoDELLA* to understand how GA controls plant development. This study helps to understand the functions of DELLA genes in orchids, providing clues for exploring the regulatory networks that control growth and stress responses, eventually allowing the establishment of breeding programs that employ transgenic techniques to improve growth, development, productivity, and biotic or abiotic stress resistance.

The original article [1] has been corrected.
