# Supplementary material for: Uncovering the involvement of DoDELLA1-interacting proteins in development by characterizing the DoDELLA gene family in Dendrobium officinale
Source: BMC Plant Biol. 2023 Feb 13;23:93. doi: 10.1186/s12870-023-04099-w (PMC9926750; doi:10.1186/s12870-023-04099-w)
Supplement: Supplementary file 1 — Additional file 1: Figure S1. Expression analysis of DoDELLA genes in leaves of Dendrobium officinale roots and stems in response to 100 μm GA3 by qRT-PCR. Figure S2. Expression analysis of DoDELLA genes in stems and leaves under NaCl (250 mM) stress by qRT-PCR. Figure S3. Expression analysis of DoDELLA genes in stems and leaves) under drought (15% PEG) stress by qRT-PCR. Table S1. Primers used for qRT-PCR. Table S2. Primers used for subcellular localization analysis. Table S3. Primers used for the yeast two-hybrid assay. [file 12870_2023_4099_MOESM1_ESM.docx]

**Supplementary Materials**

**Uncovering the involvement of DoDELLA1-interacting proteins in development by characterizing DoDELLA gene family in *Dendrobium officinale***

Danqi Zeng^1,2,3^, Can Si^1,2^, Jaime A. Teixeira da Silva^4^, Hongyu Shi^1,2,3^, Jing Chen^1,2,3^, Lei Huang^1,2,3^, Juan Duan^1,2^ , Chunmei He^1,2*^

^1^Key Laboratory of South China Agricultural Plant Molecular Analysis and Genetic Improvement, Provincial Key Laboratory of Applied Botany, South China Botanical Garden, Chinese Academy of Sciences, Guangzhou, 510650, China

^2^ South China National Botanical Garden, Guangzhou 510650, China

^3^University of the Chinese Academy of Sciences, Beijing, 100049, China

^4^Independent Researcher, Ikenobe 3011-2, Kagawa-ken 761-0799, Japan

*****Correspondence: hechunmei2012@scbg.ac.cn (C.H.)

**
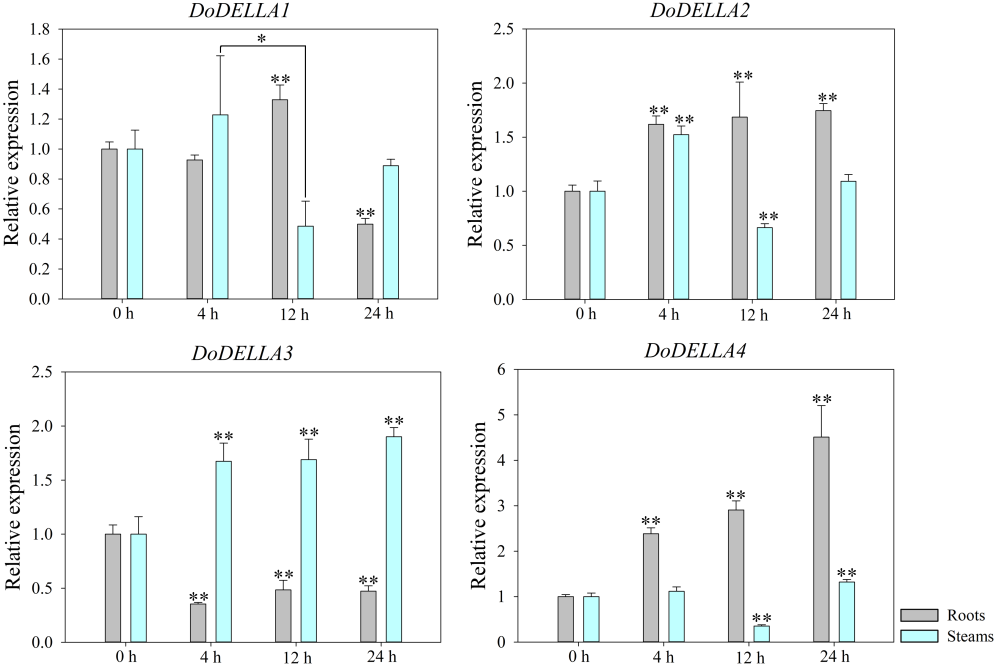
**

Figure S1. Expression analysis of *DoDELLA* genes in leaves of *Dendrobium officinale* roots and stems in response to 100 μm GA_3_ by qRT-PCR. Data represents the mean ± standard deviation (SD) of three biological replicates (n=3). Asterisks denote statistically significant differences: * *p* < 0.05; ** *p* < 0.01.


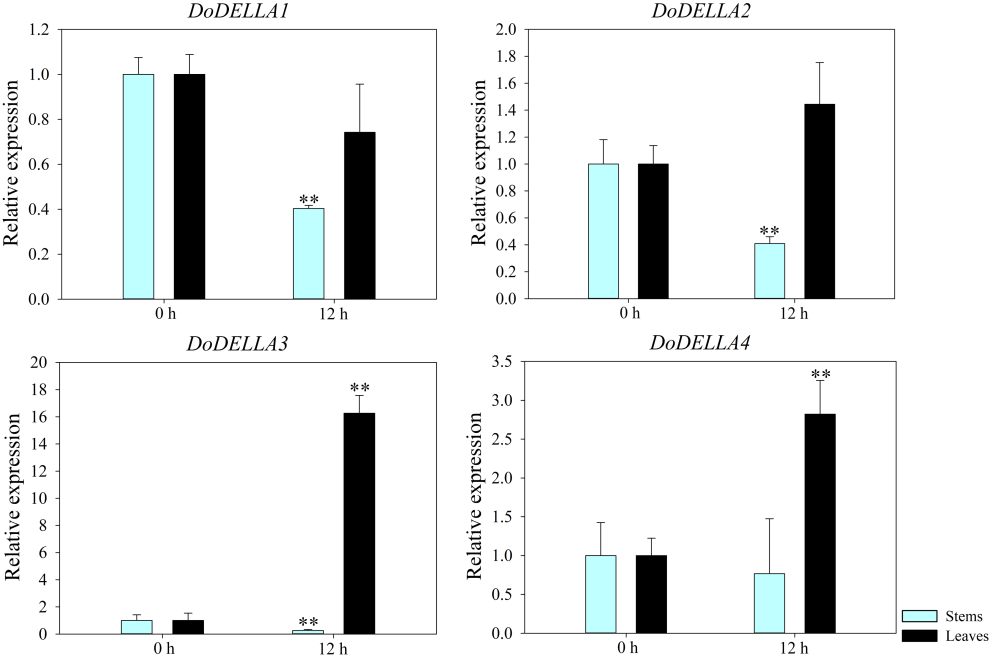


Figure S2. Expression analysis of *DoDELLA* genes in stems and leaves under NaCl (250 mM) stress by qRT-PCR. Data represents the mean ± standard deviation (SD) of three biological replicates (n=3). Asterisks denote statistically significant differences: * *p* < 0.05; ** *p* < 0.01.


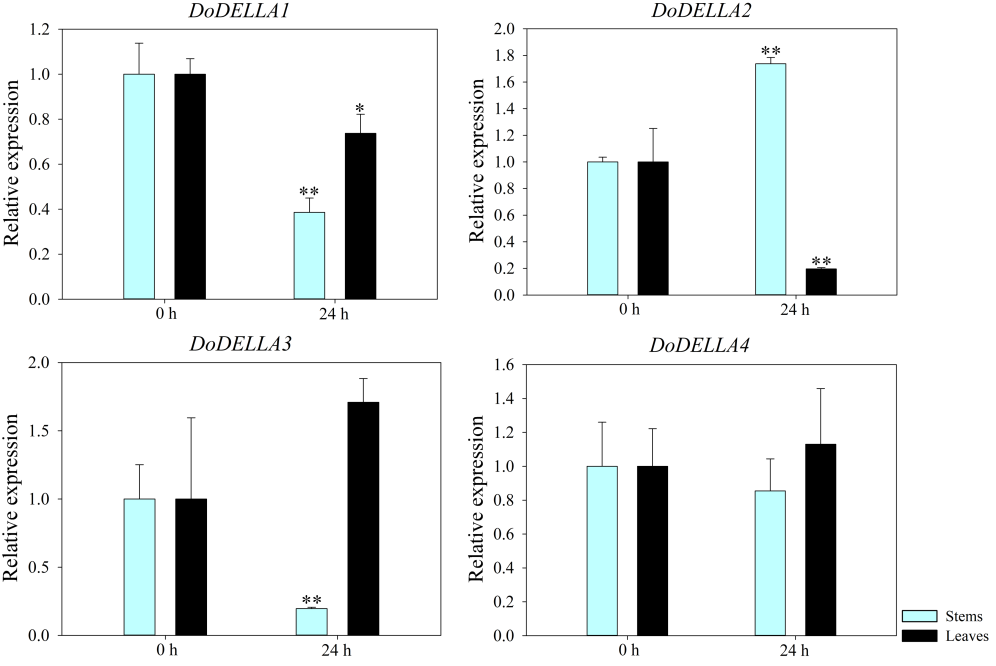


Figure S3. Expression analysis of *DoDELLA* genes in stems and leaves) under drought (15% PEG) stress by qRT-PCR. Data represents the mean ± standard deviation (SD) of three biological replicates (n=3). Asterisks denote statistically significant differences: * *p* < 0.05; ** *p* < 0.01.

**Table S1.** Primers used for qRT-PCR.

| **Primer name** | **Primer sequences (5′→3′)** |
| --- | --- |
| DoDELLA1-F | CGAGGCACTGCACTTCTATT |
| DoDELLA1-R | CCGAGATAGACTTCCGACATAAAC |
| DoDELLA2-F | CTCCTGTTGTCTTCCCTGATTT |
| DoDELLA2-R | TCGGGTTCCACTATCGATCT |
| DoDELLA3-F | CAAGAGAGTGGAAGCGGATTAG |
| DoDELLA3-R | ATCGGCATAGCTGACGAATAAA |
| DoDELLA4-F | CGGAGTCCTTGCATTACTACTC |
| DoDELLA4-R | CGAGAAATACCTCCGACATCAC |

F, forward; R, reverse

**Table S2.** Primers used for subcellular localization analysis.

| **Primer name** | **Primer sequences (5′→3′)** |
| --- | --- |
| YFP-DoDELLA1-F | AGCTCAAGCTTCGAATTCATGAAGAGGGAGAACATGGA |
| YFP-DoDELLA1-R | CCGTCGACTGCAGAATTCACGAGCATCAGAGGCCGCGG |

F, forward; R, reverse

**Table S3.** Primers used for the yeast two-hybrid assay. BD-DoDELLA1-F/R was used to clone the full length of the DoDELLA1 vector, BD-DoDELLA1-F/R1 was used to clone the truncated DoDELLA1-active domain, and BD-DoDELLA1-F1/R was used to clone the truncated DoDELLA1-GRAS domain.

| **Primer name** | **Primer sequences (5′→3′)** |
| --- | --- |
| BD-DoDELLA1-F | CATGGAGGCCGAATTCATGAAGAGGGAGAACATGGA |
| BD-DoDELLA1-R | GGATCCCCGGGAATTCACGAGCATCAGAGGCCGCGG |
| BD-DoDELLA1-F1 | CATGGAGGCCGAATTCATCCGTCTGGTTCACGCACT |
| BD-DoDELLA1-R1 | GGATCCCCGGGAATTCTCCGACCTCCTGAGTGTCCTCC |
| AD-DoMYB39-F | GGAGGCCAGTGAATTCATGGGGAGATCTCCTTGTTG |
| AD-DoMYB39-R | CACCCGGGTGGAATTCTCTGAATTCCAGCAACCCGT |
| AD-DoMYB306-F | GGAGGCCAGTGAATTCATGGGAAGAGCTCCTTGCTG |
| AD-DoMYB306-R | CACCCGGGTGGAATTCCATGAAATCACTTACATCTAGC |
| AD-DoWAT1-F | GGAGGCCAGTGAATTCATGGGAGATGAAGAAGGGAG |
| AD-DoWAT1-R | CACCCGGGTGGAATTCTTTTGGTTCATCAACATC |

F, forward; R, reverse
